# Experimental and optimised data set for hot extrusion of B_4_C/Al 6061 composite using Taguchi coupled GRA technique

**DOI:** 10.1016/j.dib.2020.105389

**Published:** 2020-03-07

**Authors:** Venkatesh Chenrayan, Mohanram Parthiban, Chandraprabu Venkatachalam, Mengistu Gelaw

**Affiliations:** School of Mechanical,Chemical and Materials Engineering, Adama Science and Technology University, Adama, Ethiopia

**Keywords:** Composite material, Taguchi's technique, Grey grade, Tensile strength, Extrusion, Extrusion force

## Abstract

Modern aluminium composites reinforced with variety of hard particles become more usage in Industrial environment. But manufacturing of composites with homogenously distributed reinforcement becomes the challenge. To overcome this challenge, most of the Aluminium composite are undergone a secondary extrusion process. The data presented here are related to hot extrusion of round geometry to hexagonal section Al/B_4_C composite. Availability of data is extended to expose the optimal parameters of the process over the extrusion load and tensile strength of the extrudate. Ram speed, geometry of die profile, billet temperature and friction within the die and billet interface have been considered as chief process parameters which influence the extrusion load and strength of the product. Totally, nine experiments were conducted as per Taguchi's L9 orthogonal array to reach optimal parameters. Most influencing parameters with ranking significance have been arrived through ANOVA, MRPI and grey grade. Optimal parameters were compared with confirmation experiments and predicted one to justify the investigation

**Table:** **Specification Table**

Subject area	Mechanical engineering
More specific subject area	Metal forming
Type of data	Table, figure and text file.
How data was acquired	After completing each trial, extrusion load and tensile strength was observed through Universal Testing Machine 600 KN capacity UTE-60
Data format	Raw and analysed
Parameters for data collection	The extrusion process parameters such as ram speed of 4, 8 and 12 mm/min, Die profiles of conical, fillet radius and cosine, billet temperature of 200 °C, 300 °C and 400 °C, friction factor of 0.2. 0.6 and 0.8 was maintained to complete the planned nine experiments.
Description of data collection	35 µm size of boron carbide particles with 15% volume fraction was employed to reinforce with the Al 6061 aluminium alloy to develop the Al/B_4_C composite through stir casting route. Stirrer motor was set at 700 rpm to achieve uniform mixture. During extrusion, extrusion die with die gland of conical, fillet radius and cosine profiled geometry, were utilised for the experiment. The friction factors were controlled by applying a solid lubricant, graphite powder within the die billet interface. Fully lubricated by applying with graphite powder as level1, partially lubricated with graphite powder considered as level 2 and zero lubrication, dry extrusion as level 3 were maintained to investigate the effect of friction. For each trial, extrusion load and tensile strength were observed as raw data and is shown in Table 8
Data source location	Adama Science and Technology University, Adama, Ethiopia
Data accessibility	Data are provided in this article and supplementary file
Related article	Venkatesh.C, Venkatesan.R. Design and analysis of stream lined extrusion die for round to hexagon through Area mapping technique, Upper bound technique and Finite element method. *Journal of Mechanical Science and Technology.* 28(5) (2014) 1867–1874. https://doi.org/10.1007/s12206–014–0136–0

## Value of the data

• The data finds a pivotal role to demonstrate the optimum process conditions of hot extrusion not only for extruding Al/B_4_C composite but also for all materials.
• The data are found useful to fellow researchers and industrialist to know the intense effect of three chief extrusion process parameters over the extrusion load.
• The data can be useful to compare further optimisation of processes through any of the conventional or unconventional way of optimisation technique for the hot extrusion process.
• The data presented here are much more beneficial for the design and development of extrusion dies.

## 1 Data description

**Fig. 1:**
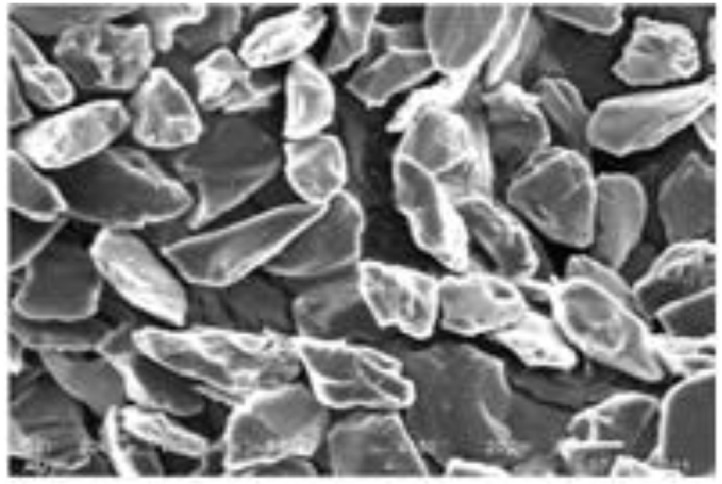
SEM image of B_4_C particles.

**Fig. 2:**
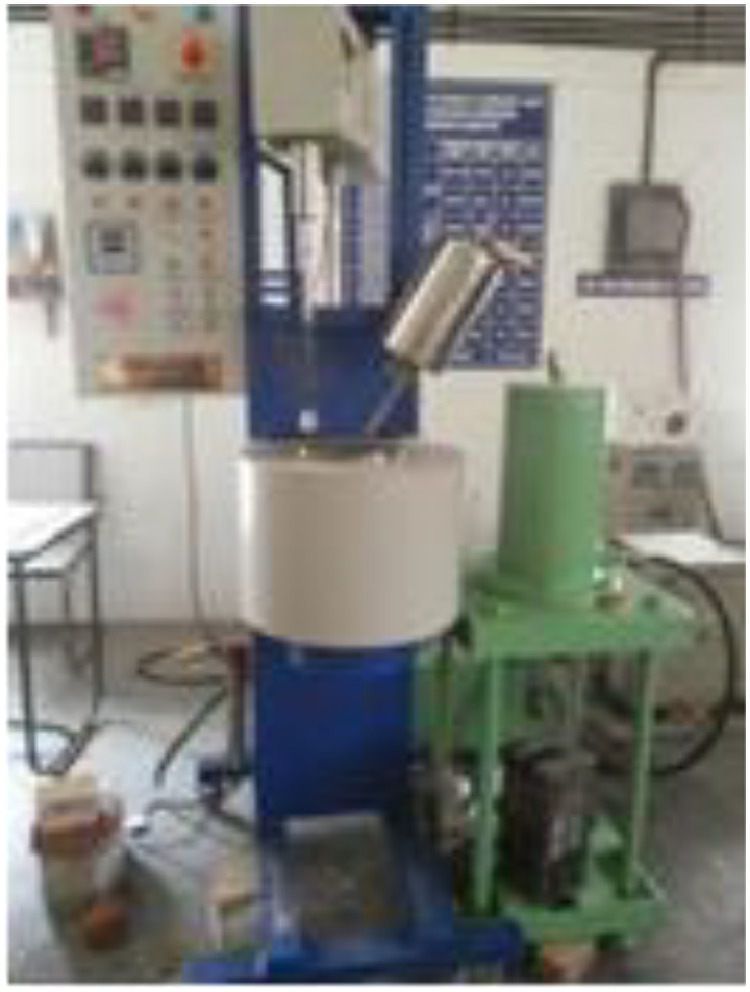
Stir casting setup.

**Fig. 3:**
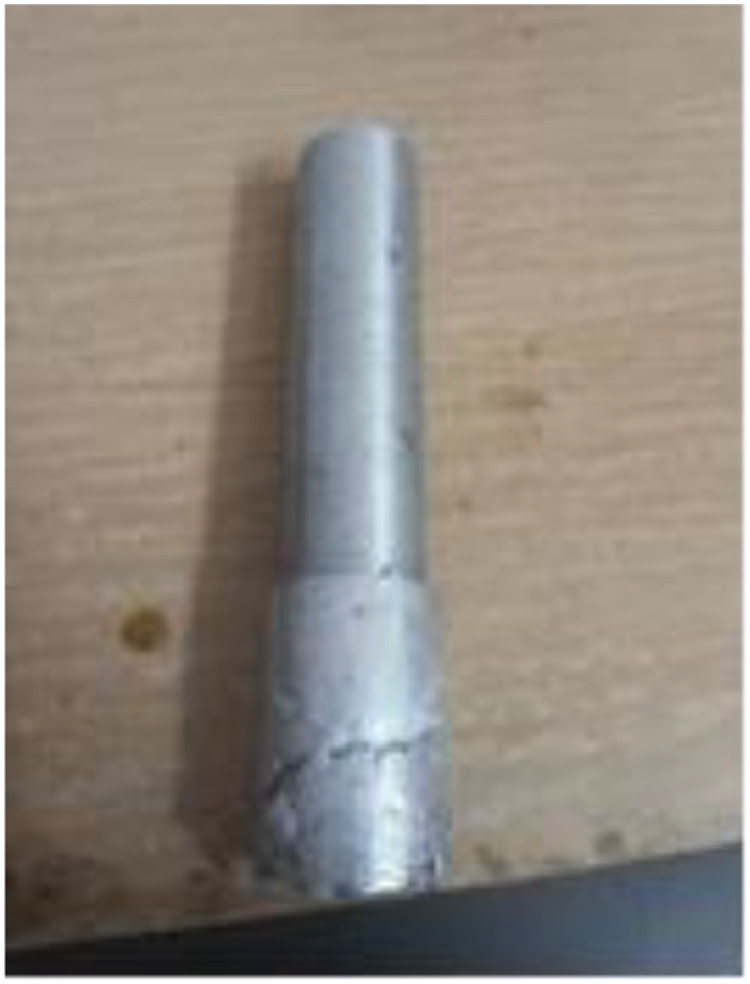
Al–B_4_C composite.

**Fig. 4:**
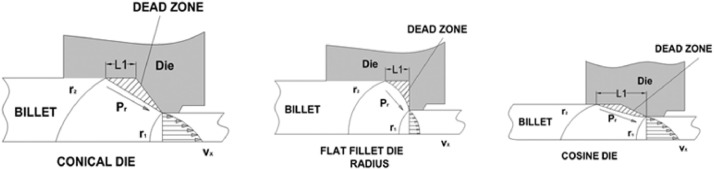
Three different die profile.

**Fig. 5:**
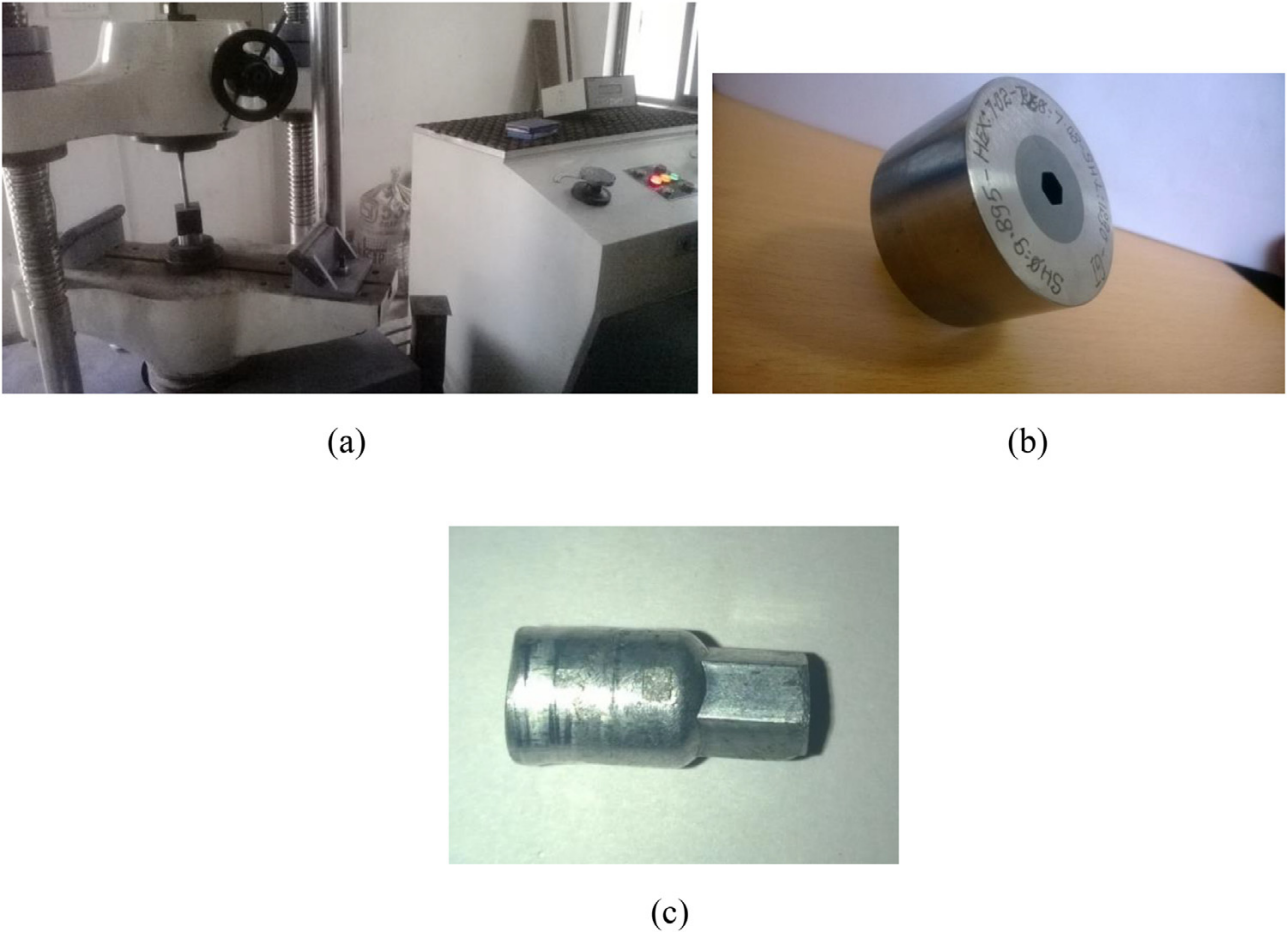
a) Experimental resource. b) Extrusion die. c) Extrudate.

**Fig. 6:**
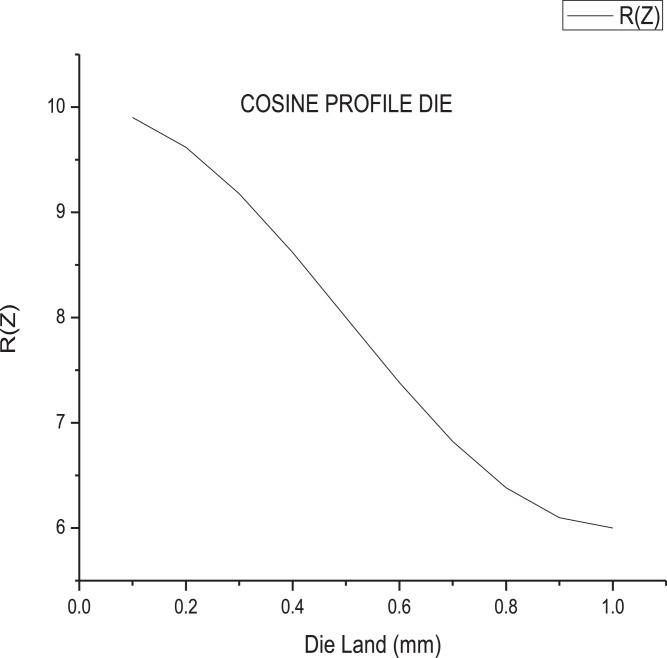
Cosine profile.

**Fig. 7:**
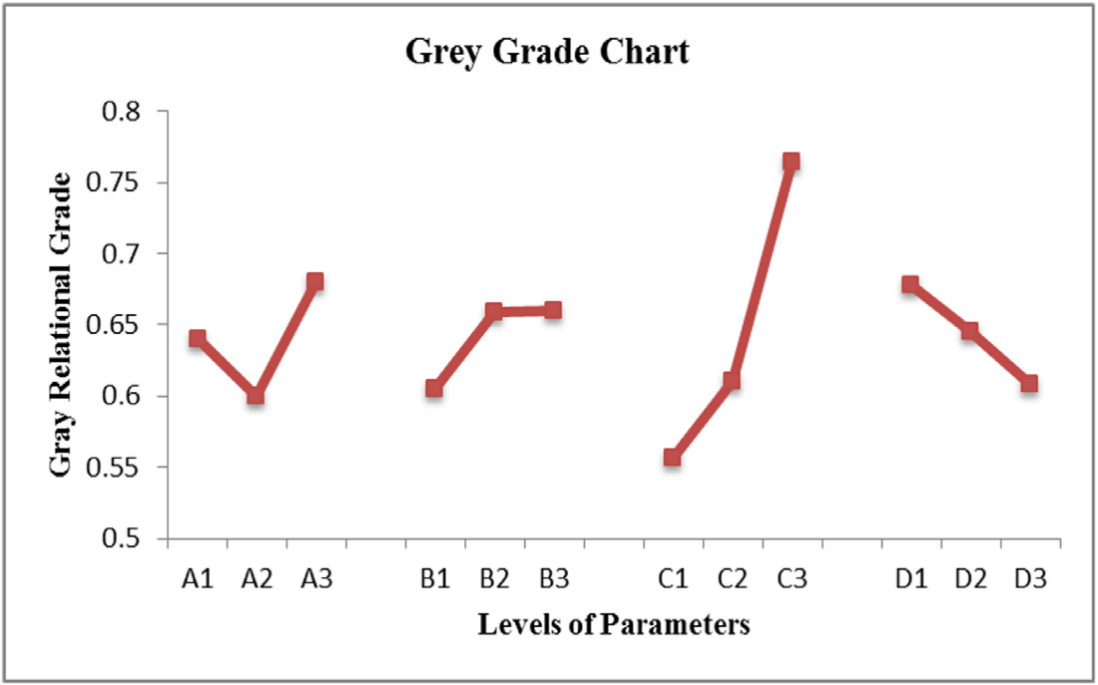
Grey grade chart.

**Fig. 8:**
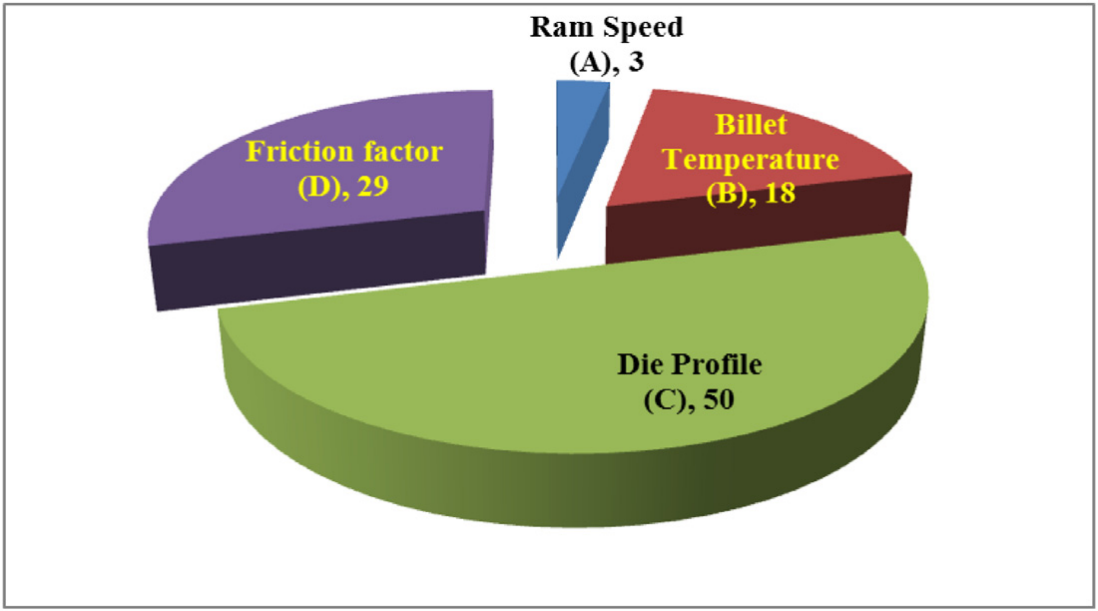
Percentage of influence of each parameter.

The data presented in this article are both raw and analysed in natures which are related to hot extrusion process of Aluminium 6061 alloy reinforced with boron carbide particles as shown in Fig. 1. Table 1 shows the specification details of Scanning Electron Microscope employed for the experiment. The chemical composition of Aluminium 6061 and Boron carbide are tabulated in Table 2 and Table 4, respectively. Mechanical properties of Aluminium 6061 and Boron carbide are shown in Table 3 and Table 5, respectively. Experimental condition followed for each trial has been given in Table 6. Selection of process parameters and their levels with corresponding values are given in Table 7. Observed values of responses (extrusion load and tensile strength) through nine experiments under the principle of Taguchi's L9 orthogonal array have been tabulated as a raw data in Table 8. Table 9 depicts the manipulated S/N ratios and normalised S/N ratios. Computed grey coefficients and grade has given in Table 10. The ranking effect of each parameter over the response can be known from Multi Response Performance Index which has tabulated in Table 11. The percentage of contribution over the impact of response of each parameter has been given as ANOVA Table 12. Table 13 interprets the comparison of grey grade between random and optimal parameters. Fig. 2 depicts the stir casting set up and the casted specimen is shown in Fig. 3. The schematic view of three different profiles used for experiment is shown in Fig. 4. Fig. 5(a), (b) and (c) represents the experimental setup, fabricated cosine profiled die and extrudate respectively. The geometrical profile of cosine curve is shown in Fig. 6. Pictorial view of computed grey graph is presented in Fig. 7 and the percentage of influence of each parameter is shown in Fig. 8. Raw data are provided in supplementary file

**Table 1:** SEM details.

Instrument	JSM6360
Signal	SEI
WD	27
Magnification	100
Accel Volt	23
Spot size	35

**Table 2:** Chemical compositions of a Al 6061 alloy.

Mg	Si	Fe	Cu	Cr	Mn	Zn	Ti	Al
0.84	0.65	0.23	0.22	0.22	0.03	0.1	0.01	Bal

**Table 3:** Properties of aluminium 6061.

Density	Elastic modulus	Yield strength	Thermal conductivity	Melting point	Hardness BHN
2.7 gm/cm^3^	68.9 GPa	276 MPa	167 W/mK	652 °C	95

## 2 Experimental design, materials, and methods

### 2.1 Materials

Boron carbide particles with particle size of 35 µm observed through Scanning Electron Microscope whose specification is shown in Table 1, were used to reinforce the proposed composite. Microscopic view of the particles is shown in Fig. 1. Aluminium 6061 alloy was utilised as matrix material.

### 2.2 Methods

Stir casting set up as shown in Fig. 2 was employed to fabricate the composite. Initially Aluminium alloy 6061 whose chemical composition and mechanical properties has been given in Tables 2 and 3 respectively was melted at 750  °C in a crucible, made out of graphite. Then, B_4_C particles whose chemical composition and mechanical properties has been given in Tables 4 and 5 respectively were preheated up to 500  °C and mixed with molten bath. In order to achieve a homogenous mixture of composite, stirring process with nearly 400 rpm was maintained for about 45 minutes. The mixture was poured in to the cylindrical mould having with dimensions of 12 mm diameter and 25 mm long. Fig. 3 shows the casted composite. Experimental extrusion was carried out by following the conditions given in Table 6, with the help of UTM shown in Fig. 5(a). Three extrusion dies with different die profile namely, fillet radius, conical and cosine curve, illustrated in Fig. 4 were manufactured through Die sink and wire cut EDM and one of the die manufactured with cosine profile is shown in Fig. 5(b) and typical cosine curve has been shown in Fig. 6. Initially, the casted specimen was heated to a given temperature within muffle furnace. The hot round billet was loaded in the die and then the punch was used to press the billet by following a referred level of ram speed. During this deformation, one of the responses, extrusion load was recorded. The experimental trial was carried out by following three levels of temperatures and three levels of ram speed by utilising three different dies.

**Table 4:** Chemical compositions of boron carbide.

B + C	Si	C	Fe	B_2_O_3_
Bal	0.1	0.17	0.2	0.1

**Table 5:** Properties of Boron carbide.

Density	Elastic modulus	Yield strength	Thermal conductivity	Melting point	Hardness (Vickers)
2.5 g/cm^3^	460 GPa	569 MPa	42 W/mK	2763 °C	38 GPa

**Table 6:** Experimental conditions.

Press tool	600 KN UTM (Digital) UTE-60
	Resolution – 0.1mm
	Clearance between columns – 600 mm
	Minimum test speed – 0.1 mm/min
	Ram stroke – 250 mm
	Accuracy- ± 0.5%
Material	15% vol. B_4_C/Al 6061 composite
Die	Conical, Fillet radius, Cosine profiled die geometry
Punch	10 mm dia × 50 mm length EN 28 rod
Lubrication	Graphite powder
Billet dimensions	10 mm dia × 25 mm length
Heating source	Muffle furnace

**Table 7:** Process parameters and their levels.

Symbol	Process parameters	Unit	Level 1	Level 2	Level 3
A	Ram speed	mm/min	4	8	12
B	Billet temperature	^o^C	200	300	400
C	Die profile geometry		Fillet radius	Conical	Cosine
D	Friction factor		0.2	0.6	0.8

### 2.3 Experimental design

#### 2.3.1 Selection of process variable and their levels

Extrusion is one of the important metal forming operation, which is highly influenced by geometry of the die profile, initial billet temperature, friction condition between the die and billet interface and also ram speed. These four parameters were considered to be more significant and their levels were also decided to cover low, medium and higher region of magnitudes so as to accomplish optimal parameter set,

Whenever frictional factor is a matter of concern during the experiments, die surface was fully lubricated by applying a graphite powder as higher level, partially lubricated as medium and zero lubrication or dry extrusion as low level.

#### 2.3.2 Design of experiment

Taguchi's method of experimental design can be found very much useful in solving the complex engineering problems with lean data. Implementation of orthogonal array paves the way to decrease the number of experiments drastically. Each column in OA represents important parameters which influence the responses. The degree of freedom and number of trials were decided by the number of parameters and their levels. The order of parameter level for each trial has mentioned in Table 8.

**Table 8:** Experimental layout using L9 orthogonal arrays.

Experiment no.	Extrusion process parameters								Extrusion load (tons)	Tensile strength Mpa
	Ram speed (A)		Billet temperature (B)		Die profile geometry (C)		Friction condition (D)			
	Level	Value (mm/min)	Level	Value (°C)	Level	Shape	Level	Value		
1	1	4	1	200	1	Conical	1	0.2	18.5	428
2	1	4	2	300	2	Fillet radius	2	0.6	19.1	381
3	1	4	3	400	3	Cosine	3	0.8	20.1	353
4	2	8	1	200	2	Fillet radius	3	0.8	21.7	315
5	2	8	2	300	3	Cosine	1	0.2	20.5	344
6	2	8	3	400	1	Conical	2	0.6	17.1	394
7	3	12	1	200	3	Cosine	2	0.6	20.3	348
8	3	12	2	300	1	Conical	3	0.8	19.5	369
9	3	12	3	400	2	Fillet radius	1	0.2	19.8	361

**Table 9:** S/N ratio and normalised S/N ratio.

Trial no	S/N ratio		Normalised S/N ratio	
	Extrusion load	Tensile strength	Extrusion load	Tensile strength
1	−25.34	52.62	0.328	0.000
2	−25.62	51.62	0.463	0.374
3	−26.06	50.95	0.676	0.625
4	−26.73	49.95	1.000	1.000
5	−26.23	50.73	0.758	0.707
6	−24.66	51.91	0.000	0.266
7	−26.15	50.83	0.719	0.670
8	−25.80	51.34	0.550	0.479
9	−25.93	51.15	0.613	0.550

**Table 10:** Grey relational coefficients and grey relational grade.

Trial no	Normalised S/N ratio		Quality loss		Grey relational coefficient		Grey grade
	Extrusion load	Tensile strength	∆_Extrusion load_	∆_Tensile strength_	GC_Extrusion load_	GC_Tensile strength_	
1	0.328	0.000	0.672	1.000	0.598	0.500	0.549
2	0.463	0.374	0.537	0.626	0.650	0.615	0.632
3	0.676	0.625	0.324	0.375	0.755	0.727	0.741
4	1.000	1.000	0.000	0.000	1.000	1.000	0.500
5	0.758	0.707	0.242	0.293	0.800	0.773	0.786
6	0.000	0.266	1.000	0.734	0.500	0.576	0.538
7	0.719	0.670	0.281	0.330	0.780	0.752	0.766
8	0.550	0.479	0.450	0.521	0.512	0.657	0.584
9	0.613	0.550	0.387	0.450	0.712	0.689	0.700

**Table 11:** Mean MRPI and the ranking of factors effect.

Factors	Ram speed (A)	Billet temperature (B)	Die profile geometry (C)	Friction factor (D)
Level 1	0.64	0.605	0.557	0.678*
Level 2	0.60	0.659	0.61	0.645
Level 3	0.68*	0.66*	0.764*	0.608
Max–min	0.04	0.055	0.207	0.07
Rank	4	3	1	2

**Table 12:** ANOVA table.

Source of variation	DOF	Sum of squares	Mean squares	F value	% Contribution	Rank
Ram speed (A)	2	0.0041	0.002	0.2	3	4
Billet temperature (B)	2	0.024	0.012	1.2	18	3
Die profile (C)	2	0.066	0.033	3.3	50	1
Friction factor (D)	2	0.038	0.019	1.9	29	2
Error	8	0.083				
Total	16			6.6	100

**Table 13:** Comparative table of the grey grade for the random and optimal process parameters.

Response Value	Optimal process parameters	
	Predicted A_3_B_3_C_3_D_1_	Confirmation experiment A_3_B_3_C_3_D_1_
Extrusion load (tons)		17.87
Tensile strength (MPa)		413
Grey relational grade	0.716	0.689

Whenever, the extrusion process is considered, load needed to extrude the billet becomes a significant response because it decides the press capacity [1], [2], [3]. Moreover the importance of secondary extrusion process for the processing of composite lies with homogenous distribution of reinforcement in order to enhance the strength of the composite, hence tensile strength of the composite being another objective [4]. It is most important to know the degree of influence of the extrusion parameters over these objectives with optimised way. However, inclusion of two responses changes the problem into multivariable approach. As per the Taguchi's robust design of experimental approach, there shall be nine experiments conducted based on L9 OA [5]. For each experimental trial, the extrudate shown in Fig. 5(C) was made into observation of extrusion load and tensile strength using UTM.

#### 2.3.3 Grey relational analysis

GRA can be applicable to evaluate the problem with more than one objective. The multi response optimisation can be changed in to single objective problem. The data observed through experiments were analysed and normalised between zero to one so as to generate the grey relational coefficients. Initially the response data recorded in experiment trials were transformed into S/N ratio. The effect of response in terms of larger or smaller was arrived. For the present case, one of the objective extrusion load to be as lower as possible and another objective tensile strength should be as higher as possible are preferred. S/N ratio were calculated as per smaller the better type and larger the better type approaches as follows,

(1) $$\text{For}\;\text{smaller}\;\text{the}\;\text{better}\;\text{type}\;\frac{S}{N}ratio=-10\log\left[\frac{1}{n}\sum_{i=1}^{n}y_{ij}^{2}\right]$$

(2) $$\text{For}\;\text{larger}\;\text{the}\;\text{better}\;\text{type}\;\frac{S}{N}ratio=-\log\left[\frac{1}{n}\sum_{i=1}^{n}\frac{1}{y_{ij}^{2}}\right]$$

The purpose of normalisation is to express the analysed data in to single decimal ranging from 0 to 1. The following relation was employed to execute the normalisation under smaller the better type approach.

Z_ij_ = normalised value for the *i* th experiment/trial for the *j* th response

(3) $$Z_{ij}=\frac{\max\left(Y_{ij},i=1,2,\ldots ,n\right)-Y_{ij}}{\max\left(Y_{ij},i=1,2,\ldots ,n\right)-\min\left(Y_{ij},i=1,2,\ldots ,n\right)}$$

From the normalised S/N ratio, the grey relational coefficient can be manipulated by using the following relation,

(4) $$GC_{ij}=\frac{\Delta_{\min}+\lambda \Delta_{\max}}{\Delta_{ij}+\lambda \Delta_{\max}}\left\{ \begin{array}{c} i=1,2,\ldots ,n-\exp eriments\\ j=1,2,\ldots ,m-responses \end{array}\right.$$

Where GC_ij_ = Grey Relational Coefficients for the *i* th experiment/trial for the *j* th response

∆ = Absolute difference between Y_oj_ and Y_ij_ which is a deviation from the target value and can be treated as quality loss
Y_oj_ = Ideal normalised value of the *j* th response
Y_ij_ = the *i* th normalised value of the *j* th response
∆_min_ = Minimum value of ∆
∆_max_ = Maximun value of ∆
λ = Distinguishing coefficient defined in the range 0 ≤ λ ≥ 1
The Grey Relational Grade (G_i_) can be determined with the help of following relation,
  (5) $$G_{i}\frac{1}{m}\sum GC_{i}$$

The manipulated Grey Relational grade now can be equated with Multi Response Performance Index (MRPI), so as to convert the multi objective problem into single objective. The optimal parameters required to extrude the composite with minimum extrusion load and maximum tensile strength can be earned through MRPI data.

It is quite clear from the MRPI analysis that, the effect of die profile geometry is more significant than the rest of other three parameters followed by the effect of friction. The higher values of MRPI are taken into consideration for arriving the optimal parameters. It is quite clear from the grey grade graph presented in Fig. 7 which confirms the optimal level of parameters for the extrusion process as A_3_B_3_C_3_D_1._

The main intention of constructing ANOVA table is to evaluate the quantum of significance of each parameter over the responses. The rank and percentage of contribution of each parameter can be known through ANOVA. From Fig. 8, it is very much clear that the die profile has got the most influencing characteristic over the responses by achieving highest contribution of 50% followed by friction factor as second and billet temperature as third influencing parameters. It is evident that ram speed becomes the least significant parameter in the study. The physical reason behind this influence has observed that, the nature of profile with uniform curvature of cosine profile ensures the homogeneity in plastic deformation and material flow by preventing the chances of agglomeration of B_4_C particles near the die entrance [6]. Development of more frictional effect within die and billet region makes the peripheral layer of the work material to deform much slower than the central zone. This imbalanced strain behaviour during plastic deformation obviously requires more extrusion load, which in turn shows the importance of friction factor over the extrusion process. At higher temperatures, the molecular bonding between adjacent molecules becomes diffused, which in turn causes quick deformation by absorbing minimum extrusion load than at low temperature.

## 3 Verification of optimal parameter through confirmation experiment

Confirmation experiment was carried out by following the optimised process parameters A_3_B_3_C_3_D_1_. The experiment was conducted with a ram speed of 12 mm/min, with cosine profiled die, applied with full lubrication and with a billet temperature of 400  °C. The observed values of extrusion force and tensile strength are as 17.87 tons and 413 MPa respectively.

The predicted grey relation α_predicted_ of the bio degradable nano cutting fluid can be expressed as

$$\alpha_{\mathrm{Pr}edicted}=\alpha_{m}+\sum_{i=1}^{n}\left(\alpha_{o}-\alpha_{m}\right)$$

Where α_predicted_ is the grey relation grade for the predicted parametrrs. α_m_ is the mean average of the grey relational grades. α_o_ is the average grey relational grade of the optimal level of the fluid parameters (A_3_B_3_C_3_D_1_) and ‘*n’* is the number of significant factors considered from the response table. The computed predicted grey relational grade was 0.689.

Comparison table confirms that, the difference between predicted and confirmation experiment is within the allowable value confident interval. Hence this type of statistical investigation through minimum number experiment can be very much useful in solving complex problem in extrusion Industries to arrive for the optimal process parameters over the quality and product cost.

## Appendix Supplementary materials

Image, application 1

Image, application 2
