# Supplementary material for: Experimental and optimised data set for hot extrusion of B4C/Al 6061 composite using Taguchi coupled GRA technique
Source: Data Brief. 2020 Mar 7;29:105389. doi: 10.1016/j.dib.2020.105389 (PMC7082519; doi:10.1016/j.dib.2020.105389)
Supplement: Supplementary file 2 [file mmc2.docx]

**Raw data for graphs and charts**

Dataset for graph shown in Fig.6

| S.No | Die land | R(Z) |
| --- | --- | --- |
| 1 | 0.2 | 9.618 |
| 2 | 0.4 | 8.618 |
| 3 | 0.6 | 7.38196 |
| 4 | 0.8 | 6.38196 |
| 5 | 1 | 6 |

Dataset for graph shown in Fig.6

| S.No | Levels of parameters | Value |
| --- | --- | --- |
| 1 | A1 | 0.64 |
| 2 | A2 | 0.60 |
| 3 | A3 | 0.68 |
|  |  |  |
| 4 | B1 | 0.605 |
| 5 | B2 | 0.659 |
| 6 | B3 | 0.66 |
|  |  |  |
| 7 | C1 | 0.557 |
| 8 | C2 | 0.61 |
| 9 | C3 | 0.764 |
|  |  |  |
| 10 | D1 | 0.678 |
| 11 | D2 | 0.645 |
| 12 | D3 | 0.608 |

Data set for Chart shown in Fig.8

| S.No | Parameter | Percentage of influence |
| --- | --- | --- |
| 1 | Ram Speed (A) | 3 |
| 2 | Billet Temperature (B) | 18 |
| 3 | Die Profile (C) | 50 |
| 4 | Friction factor (D) | 29 |
